# Antibody-drug conjugates in breast cancer: current evidence and future directions

**DOI:** 10.1186/s40164-025-00632-9

**Published:** 2025-03-20

**Authors:** Ning Li, Lu Yang, Zixuan Zhao, Tian Du, Gehao Liang, Na Li, Jun Tang

**Affiliations:** 1 Department of Breast Oncology, State Key Laboratory of Oncology in South China, Guangdong Provincial Clinical Research Center for Cancer, Sun Yat-sen University Cancer Center, Guangzhou, 510060 China; 2 Department of Radiotherapy, Cancer Center, Guangdong Provincial People’s Hospital, Guangdong Academy of Medical Sciences, Southern Medical University, Guangzhou, 510080 China; 3 Shantou University Medical College, Shantou University, Shantou, 515000 China

**Keywords:** Antibody-drug conjugate, Breast cancer, Trials, Combination therapy, Biomarker

## Abstract

Antibody-drug conjugates (ADCs) are a rapidly evolving class of antitumor drugs and have already revolutionized the treatment strategy of many hematologic and solid cancers. So far, trastuzumab emtansine (T-DM1), trastuzumab deruxtecan (T-DXd), sacituzumab govitecan (SG) and datopotamab deruxtecan (Dato-DXd) are the four ADCs that have been approved by US food and drug administration (FDA) in treatment of breast cancer, and SKB264 has been approved by Chinese national medical products administration (NMPA). Many ADCs for treatment of breast cancer are currently being tested in late-phase clinical trials, with several encouraging results achieved recently. However, major issues arise during the use of ADCs, including emergence of acquired resistance, occurrence of treated-related toxicities, and identification of biomarkers of response and resistance. ADCs are being increasingly tested in combination with other agents, and novel next-generation ADC development is progressing rapidly. A better understanding of the design and development of ADCs will promote ADC development for cancer treatment. In this review, we aim to provide a broad overview of the design and the recent advances of ADCs in breast cancer. We also propose several notable future directions of ADCs in treatment of breast cancer.

## Introduction

Breast cancer remains the most common malignancy and the leading cause of cancer deaths in women worldwide [1]. Breast cancer accounts for about one fourth of cancer cases and about one sixth of cancer deaths in women globally [1]. In clinical practice, breast cancer is considered to consist of four different molecular subtypes: hormone receptor-positive (consisting of luminal A, and luminal B subtypes), human epidermal growth factor receptor 2 (HER2)-positive, and triple-negative breast cancer (TNBC) [2, 3]. Surgery, chemotherapy, radiotherapy, endocrine therapy, targeted therapy and immune therapy have represented the mainstay of breast cancer treatment according to stages and molecular subtypes [4]. However, many patients with breast cancer are still suffering from metastatic and recurrent diseases, and thus, it is necessary to explore other high-efficacy low-toxicity therapies.

Antibody-drug conjugates (ADCs) represent a novel class of cancer therapeutics combining monoclonal antibodies with a cytotoxic agent (payload) through a chemical linker [5]. Practically, the concept of creating target-specific ‘magic bullets’ in the fight against human diseases was put forward by Paul Ehrlich in the early 1900s [6]. The rationale behind the design of ADCs is to enable massive delivery of a potent cytotoxic agent to tumor cells while reducing side effects, leading to improvement of the therapeutic window [7]. Therefore, compared with conventional antitumor drugs, ADCs have strengthened antitumor efficacy and improved quality of life. The improvements in the conjugation technology and the identification of novel payloads and linkers have accelerated the development of ADCs in recent years. ADCs are a rapidly evolving class of antitumor drugs and have already revolutionized the treatment strategy of several hematologic and solid cancers [8].

The first success of ADCs was in treating hematologic malignancies, with the first approval of the ADC gemtuzumab ozogamicin in 2000 for treatment of acute myeloid leukemia [9]. It is noteworthy that gemtuzumab ozogamicin was withdrawn from the market in 2010 due to toxicity issues, and only reapproved again in 2017 with a limited dosing regimen. In 2013, the first ADC approved for treatment of solid cancers was trastuzumab emtansine (T-DM1) for second-line treatment of advanced HER2-positive breast cancer [10]. To date, there are more than 10 approved ADCs for treatment of a variety of malignancies, including leukemia, lymphoma, breast cancer, urothelial cancer, non-small-cell lung cancer, gastric cancer, cervical cancer and ovarian cancer, and more than 150 ADCs are currently being tested in clinical trials [11–13]. In breast cancer, T-DM1, trastuzumab deruxtecan (T-DXd), sacituzumab govitecan (SG), and datopotamab deruxtecan (Dato-DXd) are the four ADCs that have been approved by US food and drug administration (FDA) so far, and SKB264 has been approved by Chinese national medical products administration (NMPA). However, major issues arise during the use of ADCs, including emergence of acquired resistance, occurrence of treated-related toxicities, and identification of biomarkers of response and resistance. A better understanding of the design and development of ADCs will promote ADC development for cancer treatment. In this review, we aim to provide a broad clinical overview of the recent advances of ADCs in breast cancer.

## ADC design

**Fig. 1:**
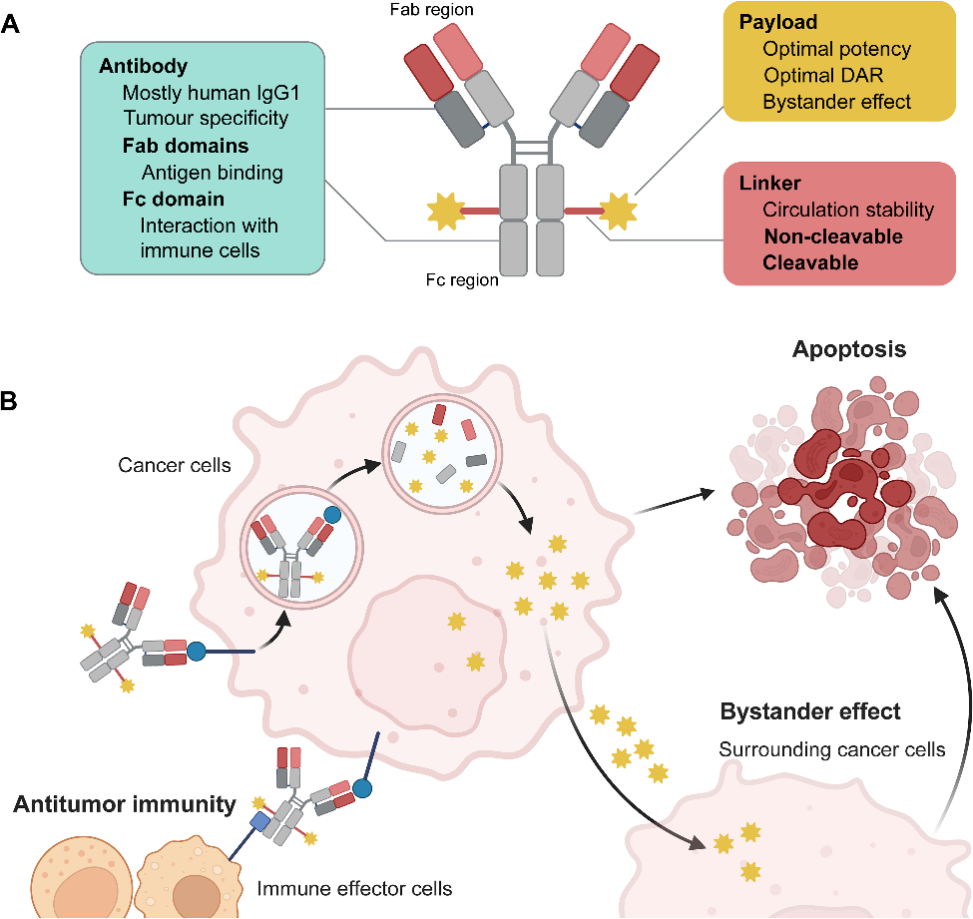
Structure and mechanisms of action of an ADC (**A**) Structure of a conventional ADC; (**B**) Mechanisms of action of a conventional ADC. ADC, antibody-drug conjugate; DAR, drug-to-antibody ratio; IgG, immunoglobulin G. Created in BioRender. Li, N. (2025) https://BioRender.com/a67b702

All classic ADCs are constructed of three major components: an antibody that targets a tumor-associated antigen, a cytotoxic payload and a linker that connects them [7] (Fig. 1A). The composition of the three components can vary tremendously between different ADCs. The canonical mechanisms of action of ADCs the following: binding to target antigens, internalization, trafficking through the endosomal/lysosomal pathway, lysosomal degradation of the antibody, intracellular payload release through linker cleavage or ADC degradation. With cleavable linkers and cell-permeable payloads, the payload can exit the target cell and act on neighboring cells (bystander effect). Moreover, the antibody portion of ADCs can also support antitumor immune responses (Fig. 1B). This is true if the antibody has wild-type Fc. Some ADCs are engineered to be ‘effector-less’ such as not to mediate any immune interaction. A better understanding of the components of ADCs will be key to developing effective and safe ADCs for cancers.

### Antibody backbones

An immunoglobulin G (IgG) is the most common antibody backbone of ADCs. IgGs are the antibody backbones because of their low molecular weight (typically 150 kDa), high affinity, long half-life, and greater tissue penetrability. Human IgGs consist of four subclasses (IgG1, IgG2, IgG3 and IgG4), and IgG1 antibodies are the now most commonly used ones in light of their ability to mediate antitumor immunity [14]. IgG2 and IgG4 antibodies have weak complement-fixation and FcγR-binding efficiencies compared to IgG1. IgG3 has been rarely used in ADC design because of its relatively short circulating half-life. Antibodies of ADCs comprise the Fab region that binds to the target antigen, and the Fc region that orchestrates multiple antitumor immune processes, including antibody-dependent cellular cytotoxicity (ADCC), complement-dependent cytotoxicity (CDC), and antibody-dependent cellular phagocytosis (ADCP) [15]. An ideal antibody recognizes antigens that are highly expressed on cancer cells but not on non-malignant cells. However, most successful breast cancer antigens including HER2 and TROP2 are also expressed on surrounding normal tissues to some extent [11]. Furthermore, an ideal antibody of ADCs should have a long half-life in the systemic circulation with low immunogenicity. In addition, the optimization of affinity of antibodies is crucial since excessively strong affinity can limit tissue penetration [16]. Thus, the antibody backbone of ADCs needs to be selected cautiously.

### Payloads

The payloads of ADCs are generally more potent than traditional chemotherapies. The payloads of current approved ADCs are mainly from one of the following classes. First, some payloads disrupt microtubules, including auristatins monomethyl auristatin E (MMAE) [17] and monomethyl auristatin F (MMAF) [18], and a maytansinoid DM1 [19]. Second, some other payloads damage DNA, including ozogamicin [20] and topoisomerase I inhibitors, such as an exatecan derivative DXd [21] and an irinotecan metabolite SN-38 [22]. Moreover, other payloads including an RNA polymerase II inhibitor amanitin [23] are currently being assessed in preclinical studies. An ideal payload of ADCs has the following features: the tumor specificity of the payload should be high; the molecular weight of the payload should be low to reduce immunogenicity; it should preferably be water-soluble to facilitate coupling with the antibody, with a functional group allowing coupling via the linker (cysteine/lysine residues, disulfide bridges); it should be stable in acidic pH so as not to be degraded in lysosomes; and its cytotoxicity must be maintained in the case of a non-cleavable linker. The drug-to-antibody ratio (DAR) refers to the average number of cytotoxic payloads attached to one antibody of an ADC. The DARs of approved ADCs range from 2 to 8. The optimal DAR for each ADC remains to be explored to balance efficacy, stability and safety.

### Linkers

Chemical linkers of ADCs connect a payload to an antibody backbone, and play a pivotal role in the efficacy and tolerability of ADCs. An ideal linker enables the payloads to remain firmly attached to the antibody in circulation, while ensuring valid release of the payloads inside the cancer cells. The linkers of ADCs can be classified as cleavable or non-cleavable [7]. Non-cleavable linkers commonly have great circulation stability, but rely on lysosomal degradation to release payloads. However, payloads with non-cleavable linkers display a reduced cell permeability and bystander effect. By contrast, cleavable linkers can be broken down by tumor-associated enzymes to release the payload [24]. Cell permeable payloads with cleavable linkers exert bystander effects, but are associated with off-target toxicities. For ADCs of breast cancer, the thioether linker used in T-DM1 is non-cleavable, while the linkers used in T-DXd and SG are cleavable [11]. Therefore, the optimal linker should balance stability, efficacy and safety of ADCs.

### HER2-targeting ADCs

About 15–20% of patients present with breast cancer whose tumors harbor HER2 overexpression and/or amplification, and the addition of anti-HER2 therapies, including trastuzumab and pertuzumab, to chemotherapy has been the standard treatment for such patients [4]. The earliest attempt of ADCs in solid tumors was in HER2-positive breast cancer. Key clinical trials of HER2-targeting ADCs in breast cancer are summarized in Table 1.

**Table 1:** Key clinical trials of HER2-targeting ADCs in breast cancer

Trial name and year of main publication	Phase	Patients (*n*)	Interventions	Efficacy outcomes (months or %)
**In advanced setting**				
EMILIA [10, 25] 2012	III	Pre-treated HER2+ ABC (*n* = 991)	T-DM1 vs. lapatinib plus capecitabine	**mPFS**: 9.6 vs. 6.4 (*P* < 0.001) **mOS**: 29.9 vs. 25.9 (*P* < 0.001) **ORR**: 43.6% vs. 30.8%
TH3RESA [26, 27] 2014	III	Pre-treated HER2+ ABC (*n* = 602)	T-DM1 vs. TPC	**mPFS**: 6.2 vs. 3.3 (*P* < 0.0001) **mOS**: 22.7 vs. 15.8 (*P* = 0.0007) **ORR**: 31% vs. 9%
MARIANNE [31, 107] 2017	III	Untreated HER2+ ABC (*n* = 1095)	T-DM1 ± P vs. TH	**mPFS**: T-DM1 vs. TH: 14.1 vs. 13.7 (*P* = 0.31); T-DM1 + P vs. TH: 15.2 vs. 13.7 (*P* = 0.14) **mOS**: 53.7 vs. 51.8 vs. 50.9 **ORR**: 59.7% vs. 64.2% vs. 67.9%
KAMILLA [28, 132] 2019	III	Pre-treated HER2+ ABC (*n* = 2002)	T-DM1	**mPFS**: 6.9 **mOS**: 27.2 **ORR**: 29.2%
KATE2 [30] 2020	II	Pre-treated HER2+ ABC (*n* = 202)	T-DM1 + atezolizumab vs. T-DM1	**mPFS**: 8.2 vs. 6.8 (*P* = 0.33)
DESTINYBreast01 [38, 39] 2020	II	Pre-treated HER2+ ABC (*n* = 184)	T-DXd	**mPFS**: 19.4 **mOS**: 29.1 **ORR**: 62.0%
DESTINYBreast03 [41–43] 2022	III	Pre-treated HER2+ ABC (*n* = 524)	T-DXd vs. T-DM1	**mPFS**: 29.0 vs. 7.2 (HR: 0.30) **mOS**: 52.6 vs. 42.7 (HR: 0.73) **ORR**: 78.9% vs. 36.9%
DESTINYBreast04 [46] 2022	III	Pre-treated HER2-low ABC (*n* = 557)	T-DXd vs. TPC	**mPFS**: 9.9 vs. 5.1 (*P* < 0.001) **mOS**: 23.4 vs. 16.8 (*P* = 0.001) **ORR**: 52.3% vs. 16.3%
TUXEDO-1 [51] 2022	II	Pre-treated HER2-low ABC with BM (*n* = 15)	T-DXd	**Overall intracranial response rate**: 73.3%, including complete intracranial response in 13.3%
DESTINYBreast02 [44] 2023	III	Pre-treated HER2+ ABC (*n* = 608)	T-DXd vs. TPC	**mPFS**: 17.8 vs. 6.9 (*P* < 0.001) **mOS**: 39.2 vs. 26.5 (*P* = 0.002) **ORR**: 69.7% vs. 29.2%
DAISY [48] 2023	II	Pre-treated HER2overexpressing/low/negative ABC (*n* = 186)	T-DXd	**ORR**: HER2-overexpressing 70.6%, HER2-low 37.5%, HER2 non-expressing 29.7%
DESTINYBreast06 [47] 2024	III	Pre-treated HER2-low or HER2-ultralow ABC (*n* = 866)	T-DXd vs. TPC	**mPFS for HER2-low disease**: 13.2 vs. 8.1 (HR: 0.62) **mPFS for HER2-ultralow disease**: 13.2 vs. 8.3 (HR: 0.78) **ORR**: HER2-low 56.5% vs. 32.2%, HER2-ultralow 61.8% vs. 26.3%
DESTINYBreast12 [53]	IIIB/IV	Pre-treated HER2+ ABC with or without BM (*n* = 504)	T-DXd	**12-month PFS in the BM cohort**: 61.6% **12-month CNS PFS in the BM cohort**: 58.9% **ORR**: 51.7% in the BM cohort vs. 62.7% in the non-BM cohort
**In (neo)adjuvant setting**				
KRISTINE [33, 133] 2018	III	HER2+ EBC (*n* = 444)	T-DM1 + P vs. TCbHP	**pCR rate: 44.4% vs. 55.7% (*p* = 0.016) 3-year iDFS**: 93.0% vs. 92.0%
KATHERINE [32] 2019	III	HER2+ EBC without pCR after neoadjuvant therapy (*n* = 1486)	T-DM1 vs. trastuzumab	**3-year iDFS**: 88.3% vs. 77.0% (*P* < 0.001)
PREDIX [134] 2021	II	HER2+ EBC (*n* = 202)	T-DM1 vs. THP	**pCR rate**: 43.9% vs. 45.5% (*p* = 0.82)
KAITLIN [34] 2022	III	HER2+ EBC (*n* = 1846)	AC-T-DM1 + P vs. AC-THP	**3-year iDFS**: 93.1% vs. 94.2%
TALENT [135] 2023	II	HR+/HER2-low EBC (*n* = 58)	T-DXd ± anastrozole	**ORR**: 75% (T-DXd alone) vs. 63% (T-DXd plus anastrozole)
WSG-ADAPT-TP [136] 2023	II	HR+/HER2+ EBC (*n* = 375)	T-DM1 ± ET vs. H + ET	**5-year iDFS**: 88.9% vs. 85.3% vs. 84.6% (*P* = 0.608) **5-year OS**: 97.2% vs. 96.4% vs. 96.3% (*P* = 0.534)
ATEMPT [36] 2024	II	HER2+ stage I breast cancer (*n* = 512)	T-DM1 vs. TH	**5-year iDFS**: 97.0% vs. 91.1% **5-year OS**: 97.8% vs. 97.9%

Abbreviations: HER2+, human epidermal growth factor receptor 2-positive; ADCs, antibody-drug conjugates; ABC, advanced-stage breast cancer; T-DM1, trastuzumab emtansine; TPC, treatment of physician’s choice; mPFS, median progression-free survival; mOS, median overall survival; ORR, objective response rate; P, pertuzumab; TH, taxane plus trastuzumab; T-DXd, trastuzumab deruxtecan; BM, brain metastases; CNS, central nervous system; EBC, early-stage breast cancer; TCbHP, docetaxel-carboplatin-trastuzumab-pertuzumab; iDFS, invasive disease-free survival; pCR, pathologic complete response; AC, anthracycline chemotherapy; ET, endocrine therapy

### Trastuzumab emtansine (T-DM1)

T-DM1 is the first anti-HER2 ADC composed of a humanized anti-HER2 antibody trastuzumab connected through a non-cleavable linker to a cytotoxic microtubule inhibitor DM1, with a DAR of 3.5. The approval of T-DM1 in treatment of HER2-positive breast cancer in 2013 was based on the results of the pivotal phase III EMILIA trial, which compared T-DM1 to lapatinib plus capecitabine in 991 patients with HER2-positive advanced breast cancer who had been previously treated with trastuzumab and a taxane [10]. The median progression-free survival (PFS) was 9.6 months in the T-DM1 group and 6.4 months in the lapatinib-capecitabine group (hazard ratio, HR, 0.65, *P* < 0.001), and the median overall survival (OS) was 30.9 months and 25.1 months (HR, 0.68, *P* < 0.001), respectively. Besides, T-DM1 was associated with not only a significant survival benefit but also a more favorable safety profile. The rates of grade ≥ 3 adverse events (AEs) were lower with T-DM1 than with lapatinib-capecitabine (41% vs. 57%). Thrombocytopenia (12.9%) and elevated concentrations of aspartate aminotransferases (4.3%) were the most commonly reported grade ≥ 3 AEs in the T-DM1 group, whereas diarrhea (20.7%) and palmar-plantar erythrodysesthesia (16.4%) were the most commonly reported grade ≥ 3 AEs in the lapatinib–capecitabine group [10]. The final OS analysis of the EMILIA trial confirmed the OS benefit (29.9 months with T-DM1 vs. 25.9 months with the control) and the favorable safety profile with T-DM1 [25]. These results confirmed the role of T-DM1 in second-line treatment of advanced HER2-positive breast cancer.

Since then, efforts have been made to expand the role of T-DM1 to post-2nd line, first-line, and (neo)adjuvant treatment. In the third-line and beyond settings, T-DM1 was associated with prolonged PFS (median, 6.2 months vs. 3.3 months) and OS (median, 22.7 months vs. 15.8 months), and a low incidence of grade ≥ 3 AEs (40% vs. 47%), compared with the treatment of physician’s choice in the phase III TH3RESA trial [26, 27]. In the KAMILLA trial, T-DM1 showed its efficacy and acceptable safety for patients with HER2-positive breast cancer and brain metastasis. In the 398 patients with baseline brain metastasis from the KAMILLA trial, the median PFS and OS were 5.5 and 18.9 months, respectively [28]. In the exploratory analysis of EMILIA for patients with asymptomatic central nervous system (CNS) metastases at baseline, T-DM1 was associated with significantly prolonged OS compared to capecitabine-lapatinib, although PFS did not differ (5.9 vs. 5.7 months) [29]. T-DM1 was also tested in combination with an anti-programmed death-ligand 1 (PD-L1) antibody atezolizumab as second-line treatment in HER2-positive advanced breast cancer in the KATE2 trial, which suggested that the addition of atezolizumab to T-DM1 did not improve PFS and was associated with more AEs [30]. T-DM1 has been tested in the first-line setting in the phase III MARIANNE trial, and the results demonstrated that the first-line treatment of T-DM1 with or without pertuzumab did not show its superiority in PFS or OS compared with the standard trastuzumab–plus–a–taxane regimen, although T-DM1 was associated with fewer AEs than trastuzumab plus a taxane [31].

For early-stage HER2-positive breast cancer, the phase III KATHERINE trial compared 14 cycles of T-DM1 and 14 cycles of trastuzumab as adjuvant therapy in patients without pathologic complete response (pCR) after neoadjuvant therapy [32]. The adjuvant treatment of T-DM1 was associated with a 50% reduction in the risk of recurrence of invasive disease or death compared with trastuzumab for HER2-positive early-stage breast cancer patients who had residual disease after neoadjuvant therapy [32]. Although adjuvant T-DM1 was established for non-pCR disease after neoadjuvant therapy, the attempt to replace trastuzumab with T-DM1 in the dual anti-HER2 regimens as neoadjuvant or adjuvant treatment was always unsuccessful. In the phase III KRISTINE trial comparing T-DM1-pertuzumab and docetaxel-carboplatin-trastuzumab-pertuzumab (TCbHP) as neoadjuvant therapy for HER2-positive stage II–III operable breast cancer, the results showed that T-DM1-pertuzumab provided a lower pCR rate than the four-drug regimen (44.4% vs. 55.7%, *P* = 0.016), although T-DM1-pertuzumab was also associated with a lower rate of grade ≥ 3 AEs (13% vs. 64%) [33]. In the phase III KAITLIN trial in patients with resected high-risk HER2-positive early-stage breast cancer, T-DM1-pertuzumab was found to have no significant iDFS advantage compared to taxane-trastuzumab-pertuzumab (THP) following anthracycline-based chemotherapy [34]. Thus, these trials failed to show superiority of T-DM1 over trastuzumab and chemotherapy for trastuzumab-naive patients. The attempt to replace paclitaxel-trastuzumab with T-DM1 as adjuvant therapy for patients with stage I HER2-positive breast cancer was explored in the phase II ATTEMPT trial, which showed that adjuvant T-DM1 for 1 year provided a favorable 5-year invasive disease-free survival (iDFS) of 97.0% [35, 36]. As the first ADC in treating solid cancers, T-DM1 is still playing a crucial role in the treatment of HER2-positive breast cancer.

### Trastuzumab deruxtecan (T-DXd, DS-8201)

T-DXd is a novel anti-HER2 ADC composed of trastuzumab conjugated to deruxtecan (DXd) [21]. Compared with T-DM1, the main novelty of T-DXd is related to its cleavable tetrapeptide-based linker, which can be selectively cleaved by cathepsins that are upregulated in tumors. This feature elevates the concentration of DXd in cancer cells, and limits systemic exposure of T-DXd, thus leading to increased efficacy in theory. Another novelty of T-DXd is the topoisomerase I inhibitor payload DXd, which is a derivative of exatecan. Additionally, the DAR of T-DXd is as high as 8 [37]. The cleavable linker and cell permeability of DXd enables the DXd to permeate to tumor microenvironment and exert a bystander effect, which is an important property of T-DXd in dealing with tumors with HER2 heterogeneity.

T-DXd was firstly evaluated in heavily pre-treated patients with metastatic HER2-positive breast cancer in the phase II DESTINY-Breast01 trial, which showed that T-DXd resulted in notable efficacy, with a median PFS of 19.4 months, a median OS of 29.1 months, and an objective response rate (ORR) of 62.0% [38, 39]. A total of 53.8% of the patients had one or more grade ≥ 3 AEs. Of note, interstitial lung disease/pneumonitis occurred in 15.8% of the patients, 2.7% of whom had treatment-related death [39]. The results of the DESTINY-Breast01 trial led to the first approval of T-DXd in 2019 for patients with HER2-positive advanced breast cancer who have received two or more anti-HER2-based regimens [40].

Subsequently, the phase III DESTINY-Breast03 trial was designed to compare T-DXd head to head with T-DM1 in patients with HER2-positive advanced breast cancer previously treated with trastuzumab and a taxane [41, 42]. The results showed that T-DXd not only improved PFS (median, 29.0 vs. 7.2 months) but also led to an OS benefit (median, 52.6 vs. 42.7 months), suggesting T-DXd is a more efficacious anti-HER2 ADC than T-DM1 [41–43]. The ORR was also significantly increased with T-DXd compared with T-DM1 (78.9% vs. 36.9%). However, grade ≥ 3 AEs occurred more frequently in the T-DXd group than the T-DM1 group (58.0% vs. 52.1%), with interstitial lung disease/pneumonitis occurring in 16.7% of the patients in the T-DXd group compared with 3.4% of the patients in the T-DM1 group, although there were no grade 4 or 5 events of interstitial lung disease/pneumonitis in either group. Based on the results of DESTINY-Breast03, T-DXd is considered to be the preferable treatment in the second-line setting for patients with advanced HER2-positive breast cancer. In the phase III DESTINY-Breast02 trial, the comparison of T-DXd with the treatment of physician’s choice for patients who had progression after prior T-DM1 indicated that ORR (70% vs. 29%), PFS (17.8 vs. 6.9 months) and OS (39.2 vs. 26.5 months) were significantly improved with T-DXd [44]. The most common grade ≥ 3 AEs were neutrophil count decrease (11% with T-DXd vs. 2% with the treatment of physician’s choice), anemia (8% vs. 3%), neutropenia (8% vs. 2%), and nausea (7% vs. 3%). These results suggested that T-DXd can overcome resistance to T-DM1. The DESTINY-Breast05 trial (NCT04622319) is currently ongoing to challenge T-DM1 with T-DXd as adjuvant therapy for patients without pCR after neoadjuvant therapy. In the neoadjuvant setting, the DESTINY-Breast11 trial (NCT05113251) is evaluating whether T-DXd alone or T-DXd followed by THP can replace the standard TCbHP regimen.

Anti-HER2 therapies have been the backbone for treating breast cancer patients who are HER2-positive, which is defined according to the HER2 over-expression (score 3+) on immunohistochemistry (IHC) or ERBB2 gene amplification on an in situ hybridization (ISH) assay. However, about 45-55% of HER2-negative breast tumors still have detectable HER2 protein (IHC 1 + or 2 + with ISH-), which is defined as HER2-low [45]. HER2-low breast cancer accounts for about two thirds of hormone receptor-positive and one third of triple-negative breast cancer. In the landmark DESTINY-Breast04 trial assessing patients with HER2-low metastatic breast cancer who had received one or two previous lines of chemotherapy, T-DXd showed significant improvements in PFS (9.9 months vs. 5.1 months) and OS (23.4 months vs. 16.8 months) compared with the chemotherapy of physician’s choice [46]. With regard to toxicity, T-DXd led to a lower rate of grade ≥ 3 AEs than the control (52.6% vs. 67.4%), and interstitial lung disease or pneumonitis occurred in 12.1% of the patients with T-DXd. These results underlined the heterogeneity of HER2 expression and resulted in the approval of T-DXd for patients with HER2-low breast cancer in 2022. Recently, the results from the DESTINY-Breast06 trial aimed to expand the role of T-DXd in HR-positive breast cancer with ultralow HER2 expression (IHC 0 with membrane staining). The results showed that T-DXd not only improved PFS for HER2-low advanced breast cancer (median, 13.2 months with T-DXd vs. 8.1 months with chemotherapy), but also improved PFS for HER2-ultralow advanced breast cancer, compared with chemotherapy (median, 13.2 months with T-DXd vs. 8.3 months with chemotherapy), suggesting T-DXd is efficacious in HER2-low/ultralow breast cancer [47]. The rate of grade ≥ 3 AEs was 52.8% with T-DXd and 44.4% with chemotherapy, with interstitial lung disease/pneumonitis occurring in 11.3% of the patients in the T-DXd group [47]. The results from the DESTINY-Breast06 trial resulted in the approval of T-DXd for patients with advanced HER2-ultralow breast cancer recently. The DAISY trial demonstrated that the ORRs were 70.6%, 37.5%, and 29.7% for HER2-overexpressing, HER2-low, and HER2-zero tumors, suggesting HER2 expression is a determinant of T-DXd response, and other mechanisms of action may be involved [48].

A substantial portion of HER2-positive breast cancer patients develop brain metastases [49]. T-DXd also shows its potent intracranial activity in patients with HER2-positive breast cancer and brain metastases. The subgroup analysis of the DESTINY-Breast01 trial suggested that single-agent T-DXd led to improved patient outcomes, with an ORR of 58.3% and a median PFS of 18.1 months [50]. In the single-arm phase 2 TUXEDO-1 trial, the T-DXd treatment was associated with an overall intracranial response rate of 73.3% for patients with HER2-positive breast cancer with brain metastases [51]. In the DEBBRAH trial, the intracranial ORR in patients with active brain metastases was 46.2% [52]. T-DXd also showed encouraging intracranial activity in patients with brain metastases in the DESTINY-Breast03 trial. The median PFS was 15.0 months with T-DXd and 3.0 months with T-DM1, and the intracranial ORR was 67.5% for T-DXd and 34.3% for T-DM1. In the DESTINY-Breast12 trial evaluating the role of T-DXd in patients with advanced HER2-potitive breast cancer with or without brain metastases, the 12-month PFS was 61.6% and 12-month CNS PFS was 58.9% for patients with brain metastases [53]. The results from DESTINY-Breast12 demonstrated the substantial intracranial activity of T-DXd in HER2-positive breast cancer. To date, to my knowledge, there is no preclinical or clinical pharmacological data of T-DXd in brain or cerebrospinal fluid. We do not know if the efficacy of T-DXd is due to its brain penetration.

### TROP2-targeting ADCs

Triple-negative breast cancer (TNBC) accounts for about 15-20% of all breast cancer, characterized by high aggressiveness, high possibility of metastasis, and poor long-term outcomes [4]. Since lacking specific targets and the heterogeneity of TNBC, developing new treatment regimens for TNBC is an unmet demand. Trophoblast cell surface antigen-2 (TROP2) is a type I cell surface glycoprotein, also known as tumor-associated calcium signal transducer 2 (TAC-STD2), membrane component chromosome 1 surface marker 1 (M1S1), and gastrointestinal tumor-associated antigen GA7331 [54]. TROP2 is relatively highly expressed in tumor tissue compared with normal tissues [55]. Accumulating evidence shows the overexpression of TROP2 in a variety of tumors, including TNBC, lung cancer, and colon cancer [54]. The earliest attempt of TROP2 ADCs in breast cancer was in TNBC. Key clinical trials of TROP2-targeting ADCs in breast cancer are summarized in Table 2.

**Table 2:** Key clinical trials with final or preliminary results of TROP2-targeting ADCs in breast cancer

Trial name and year of main results	Phase	Patients (*n*)	Interventions	Efficacy outcomes (months or %)
**In advanced setting**				
IMMU-132-01 [57, 62] 2019	I-II	Pre-treated ABC (108 TNBC; 54 HR+/HER2-)	SG	**mPFS**: 5.5; 5.5 **mOS**: 13.0; 12.0 **ORR**: 33.3%; 31.5%
ASCENT [58, 60] 2021	III	Pre-treated advanced TNBC (*n* = 468)	SG vs. TPC	**mPFS**: 5.6 vs. 1.7 (*P* < 0.001) **mOS**: 12.1 vs. 6.7 (*P* < 0.001) **ORR**: 35% vs. 5%
TROPiCS-02 [63, 64] 2022	III	Pre-treated HR+/HER2- ABC (*n* = 543)	SG vs. TPC	**mPFS**: 5.5 vs. 4.0 (*P* < 0.001) **mOS**: 14.4 vs. 11.2 (*P* = 0.02) **ORR**: 21% vs. 14%
BEGONIA [71] 2023	Ib/II	Pre-treated advanced TNBC (*n* = 62 with Dato + Durva)	Dato-DXd + Durva	**mPFS**: 13.8 **ORR**: 79%
EVER-132-001 [61] 2023	II	Pre-treated advanced TNBC (*n* = 80)	SG	**mPFS**: 5.5 **ORR**: 38.8%
KL264-01 [75] 2023	I/II	Pre-treated HR+/HER2- ABC (*n* = 41)	SKB264	**mPFS**: 11.1 **ORR**: 36.8%
TROPION-Breast01 [69, 70]	III	Pre-treated advanced HR+/HER2- ABC (*n* = 732)	Dato-DXd vs. chemotherapy	**mPFS**: 6.9 vs. 4.9 (*P* < 0.001) **ORR**: 36.4% vs. 22.9%
OptiTROP-Breast01 [76] 2024	III	Pre-treated advanced TNBC (*n* = 263)	SKB264 vs. TPC	**mPFS**: 5.7 vs. 2.3 (*P* < 0.001) **mOS**: NR vs. 9.4 (*P* < 0.001) **ORR**: 43.8% vs. 12.8%
EVER-132-002 [65] 2024	III	Pre-treated HR+/HER2- ABC (*n* = 331)	SG vs. TPC	**mPFS**: 4.3 vs. 4.2 (*P* < 0.01) **mOS**: 21.0 vs. 15.3 (*P* < 0.01) **ORR**: 20% vs. 15%
**In (neo)adjuvant setting**				
NeoSTAR [67] 2024	II	Localized TNBC (*n* = 50)	SG ± neoadjuvant chemotherapy	**pCR rate with SG**: 30% **ORR with SG**: 64%
I-SPY2.2 [72, 73] 2024	II	Localized EBC (103 with Dato-DXd; 106 with Dato-DXd plus Durva)	Dato-DXd ± Durva	**pCR rate with Dato-DXd**: 6% for HR+/HER2- vs. 30% for TNBC **pCR rate with combination**: 10% for HR+/HER2- vs. 33% for TNBC

Abbreviations: TROP2, trophoblast cell surface antigen-2; ADCs, antibody-drug conjugates; ABC, advanced-stage breast cancer; TNBC, triple-negative breast cancer; HR+, hormone receptor-positive, HER-, human epidermal growth factor receptor 2–negative; SG, sacituzumab govitecan; TPC, treatment of physician’s choice; mPFS, median progression-free survival; mOS, median overall survival; ORR, objective response rate; Dato-DXd, datopotamab deruxtecan; NR, not reached; Durva, durvalumab; EBC, early-stage breast cancer; pCR, pathologic complete response

### Sacituzumab Govitecan (SG; IMMU-132)

SG is the first-in-class TROP2-ADC, which is composed of a humanized IgG1κ anti-Trop2 antibody hRS7 connected through a cleavable CL2A linker to a topoisomerase I inhibitor SN-38 (the active metabolite of irinotecan) [11]. There are multiple unique features, including a high DAR of 7.6, a relatively potent payload SN-38, and a pH-sensitive, hydrolyzable cleavable linker, making an enhanced bystander effect in a heterogeneous environment and exhibiting a favorable therapeutic index of SG [54].

In the single-arm phase II trial assessing SG in heavily pre-treated patients with metastatic TNBC, the ORR was 30%, the median OFS was 6.0 months, and the median OS was 16.6 months [56]. The phase I/II IMMU-132-01 basket trial evaluated the efficacy and safety of SG in patients with advanced heavily pre-treated patients with solid tumors. One of the encouraging results of IMMU-132-01 was revealed in 108 patients with TNBC, where the ORR was 33%, the median PFS was 5.5 months and the median OS was 13.0 months [57]. In this trial, myelosuppression (74%), nausea (67%), and diarrhea (62%) were the most common AEs. Then, the phase III ASCENT trial compared SG with the chemotherapy of physician’s choice for patients who had relapsed disease or those who had received two or more prior standard chemotherapy regimens. Within the trial, SG was demonstrated to improve ORR (35% vs. 5%), PFS (5.6 months vs. 1.7 months) and OS (12.1 months vs. 6.7 months) [58]. With regard to toxicity, the rate of grade ≥ 3 AEs was 64% for the patients in the SG arm versus 46% for the patients in the control arm, with myelosuppression and diarrhea being more frequent with SG. However, only 5% of the patients in the SG arm discontinued the drug due to toxicities. Based on these results, SG has been the first ADC approved for TNBC patients who have received two or more prior therapies. The exploratory biomarker analysis of the ASCENT trial showed that the ORR was 44%, 38%, and 22% versus 1%, 11%, and 6% for the patients with high, medium and low TROP2 expression in the SG and control arms, and the median OS was 14.2, 14.9, and 9.3 months versus 6.9, 6.9, and 7.6 months, respectively [59]. The final results of the ASCENT trial confirmed the superiority of SG compared with chemotherapy in each TROP2 expression subgroup [60]. The above results suggested that patients with TNBC may benefit from SG regardless of TROP2 expression. The single-arm phase IIb EVER-132-001 trial confirmed the efficacy of SG in Chinese patients, with an ORR of 38.8%, a median PFS of 5.5 months and a rate of grade ≥ 3 AEs of 71.3% [61].

SG was also evaluated in patients with hormone receptor-positive (HR+)/HER2-negative (HER2-) metastatic breast cancer. In the phase I/II IMMU-132-01 trial, 54 patients with previously treated HR+/HER2- metastatic breast cancer were treated with SG. The results showed that the ORR was 31.5%, the median PFS was 5.5 months, and the median OS was 12 months. The safety profile was acceptable, with the key grade ≥ 3 AEs of neutropenia (50.0%), anemia (11.1%), and diarrhea (7.4%) [62]. Subsequently, in the phase III TROPiCS-02 trial comparing SG with chemotherapy in pre-treated, endocrine-resistant HR+/HER2- metastatic breast cancer, the median PFS was 5.5 months with SG and 4.0 months with chemotherapy [63]. The final results of the TROPiCS-02 trial showed that the median OS (14.4 vs. 11.2 months) and the ORR (21% vs. 14%) were significantly improved with SG compared to chemotherapy. Besides, the survival benefit was consistent across all TROP2 expression subgroups [64]. Grade ≥ 3 AEs occurred in 74% in the SG arm and in 60% in the chemotherapy arm. Based on the results of the TROPiCS-02 trial, SG has been the first approved ADC for HR+/HER2- metastatic breast cancer patients who have received endocrine therapy, a taxane, and a CDK4/6 inhibitor in any setting and 2–4 prior chemotherapy regimens. Recently, the EVER-132-002 confirmed the role of SG in pre-treated, endocrine-resistant HR+/HER2- metastatic breast cancer in Asian patients, with both PFS and OS (median, 21.0 with SG versus 15.3 with chemotherapy) improving with SG versus chemotherapy [65].

Since SG has been shown to have enhanced efficacy compared with chemotherapy for advanced breast cancer, researchers have tried to expand the role of SG in early-stage breast cancer. The phase III SASCIA trial is investigating the role of SG in patients with HER2- breast cancer with residual disease after standard neoadjuvant chemotherapy. The interim safety analysis showed that the safety profile of SG was manageable, with grade ≥ 3 AEs occurring in 66.7% in the SG arm and in 20.9% in the treatment of physician’s choice arm (including observation). At least one dose delay was required by 66.7% of the patients receiving SG versus 43.3% of the patients receiving capecitabine. At least one dose reduction was required by 26.7% of the patients receiving SG compared to 28.1% of the patients receiving capecitabine [66]. In the phase II NeoSTAR trial, the preliminary results showed that neoadjuvant SG alone for patients with early-stage TNBC results in a pCR rate of 30%, and an ORR of 64% [67].

### Datopotamab deruxtecan (Dato-DXd; DS-1062)

Dato-DXd is a TROP2 ADC that consists of a humanized anti-TROP2 IgG1 monoclonal antibody conjugated with a potent topoisomerase I inhibitor payload DXd via a tetrapeptide-based cleavable linker. The DAR of Dato-DXd is 4. The phase I TROPION-PanTumor01 trial evaluated the efficacy and safety of Dato-DXd in advanced solid tumors and showed promising efficacy and an acceptable safety profile for both HR+ /HER2- breast cancer and TNBC. Among the 85 patients with heavily pre-treated advanced breast cancer, 41 had HR+/HER2- disease and 44 had TNBC. The ORR and the median PFS were 26.8% and 8.3 months versus 31.8% and 4.4 months for the patients with HR+/HER2- breast cancer and TNBC, respectively [68]. The phase III TROPION-Breast01 trial compared Dato-DXd with the chemotherapy of investigator’s choice in patients with metastatic HR+/HER2- breast cancer who had received 1–2 prior lines of systemic chemotherapy [69]. The results demonstrated that Dato-DXd significantly improved PFS (median PFS, 6.9 vs. 4.9 months) and ORR (36.4% vs. 22.9%) compared to chemotherapy. Grade ≥ 3 treatment-related AEs occurred in 20.8% in the Dato-DXd arm and in 44.7% in the chemotherapy arm [69, 70]. The results of the TROPION-Breast01 trial led to the approval of Dato-DXd for patients with advanced HR+/HER2- breast cancer who have received prior endocrine-based therapy and chemotherapy recently. The phase Ib/II BEGONIA study investigated the combination of Dato-DXd and an anti–PD-L1 antibody durvalumab as first-line treatment for advanced TNBC. The results from BEGONIA showed encouraging efficacy, with an ORR of 79%, including 10% with complete response (CR) and 69% with partial response (PR). The median PFS with the combination treatment was 13.8 months. The rate of grade ≥ 3 AEs was 57% and the safety was manageable [71]. The combination of an ADC and a programmed death-1 (PD-1)/PD-L1 inhibitor has been a promising treatment regimen for TNBC.

In the phase 2 platform sequential multiple assignment randomized I-SPY2.2 trial evaluating neoadjuvant Dato-DXd for early-stage breast cancer, the investigators defined response-predictive subtypes (RPS) by a combination of gene expression signatures (response to immunotherapy and/or DNA repair deficiency) and the BluePrint assay. The pCR rate after Dato-DXd alone was 6% for HR+/HER2- disease and 30% for TNBC [72]. The pCR rates are typically very low in HR + breast cancer, and ADCs have not yet changed that. Of note, single agent Dato-DXd led to a pCR rate of 41% for hormone receptor-negative HER2^−^Immune^−^DNA repair deficiency^−^ subtype [72]. The combination of Dato-DXd and durvalumab as neoadjuvant therapy in the I-SPY2.2 trial resulted in a pCR rate of 10% for HR+/HER2- breast cancer and 33% for TNBC [73]. In the immune-positive subtype, 54% achieved pCR after Dato-DXd plus durvalumab [73]. Dato-DXd alone or with durvalumab is promising in early-stage breast cancer.

### SKB264 (MK-2870, sacituzumab tirumotecan)

SKB264 is a TROP2 ADC that shares the same monoclonal antibody as SG. Compared with SG, the main differences of SKB264 are a cleavable CL2A-based linker, and a potent belotecan-derived topoisomerase I inhibitor payload KL610023. The DAR of SKB264 is 7.4, which is similar to SG. However, the sulfonyl pyrimidine-CL2A-carbonate linker increases the stability and the bystander effect of SKB264.

In the phase I/II study investigating SKB264 for previously treated metastatic TNBC, SKB264 led to an ORR of 40%, a median PFS of 5.7 months, and a rate of grade ≥ 3 AEs of 55.9%. The most common grade ≥ 3 AEs were neutrophil count decrease (23.7%), anemia (20.3%) and platelet count decrease (16.9%) [74]. The phase I/II study assessing SKB264 for previously treated metastatic HR+/HER2- breast cancer demonstrated that the ORR was 36.8%, the median PFS was 11.1 months, and the rate of grade ≥ 3 AEs was 48.8% [75]. The phase III OptiTROP-Breast01 trial compared SKB264 with the chemotherapy of physician’s choice in patients with advanced TNBC who had received two or more prior therapies. The results revealed that the median PFS was significantly prolonged with SKB264 compared to chemotherapy (5.7 months vs. 2.3 months). The preliminary OS results also favored SKB264 (median, not reached vs. 9.4 months) [76]. The ORR was 43.8% in the SKB264 arm and 12.8% in the chemotherapy arm. Most common grade ≥ 3 AEs (SKB264 vs. chemotherapy) were neutrophil count decrease (32.3% vs. 47.0%), anemia (27.7% vs. 6.1%) and white blood cell count decrease (25.4% vs. 36.4%) [76]. In 2024, based on the favorable results from the OptiTROP-Breast01 trial, SKB264 was approved by Chinese NMPA for patients with advanced TNBC after two or more systemic therapies. Although one has to be cautious about comparing data across trials, the ORR of 43.8% and the PFS of 5.7 months of SKB264 for patients with advanced TNBC who had received two or more prior therapies were not inferior and numerically slightly better than SG, which reported the ORR of 35% and the PFS of 5.6 months.

### Other ADCs showing potential in breast cancer

Besides T-DM1 and T-DXd, multiple novel HER2-targeting ADCs have shown their potential in phase I-III trials for HER2-positive or HER2-expressing solid cancers, including trastuzumab duocarmazine (T-Duo, SYD985) [77], ARX788 [78], disitamab vedotin (RC48) [79], DHES0815A [80], FS-1502 [81], MEDI4276 [82], SHR-A1811 [83], A166 [84], and BAT8001 [85]. In the phase III TULIP trial comparing T-Duo with the therapy of physician’s choice in patients with pre-treated HER2-positive metastatic breast cancer, T-Duo demonstrated an improvement in PFS compared to the therapy of physician’s choice (35.6 vs. 32.0 months) [86]. In the phase I trial evaluating SHR-A1811 in HER2-expressing or mutated advanced solid tumors, the ORR of SHR-A-1811 for HER2-positive breast cancer was 76.3% [83].

SG and Dato-DXd are approved by the FDA, and SKB264 is approved by the Chinese NMPA. Multiple other TROP2-directing ADCs are in development. Besides SG, Dato-DXd and SKB264, other TROP2-directing ADCs that have shown their potential in clinical trials for solid cancers include BAT8008 [87], DB-1305 [88], and ESG401 [89].

In addition, novel-targeted ADCs including patritumab deruxtecan (targeting HER3) [90, 91], PF-06650808 (targeting Notch3) [92], DLYE5953A (targeting LY6E) [93], anetumab ravtansine (targeting mesothelin) [94], BMS-986,148 (targeting mesothelin) [95], PCA062 (targeting P-Cadherin) [96], and praluzatamab ravtansine (targeting CD166) [97] exhibited encouraging preliminary antitumor activity in advanced solid tumors.

**Fig. 2:**
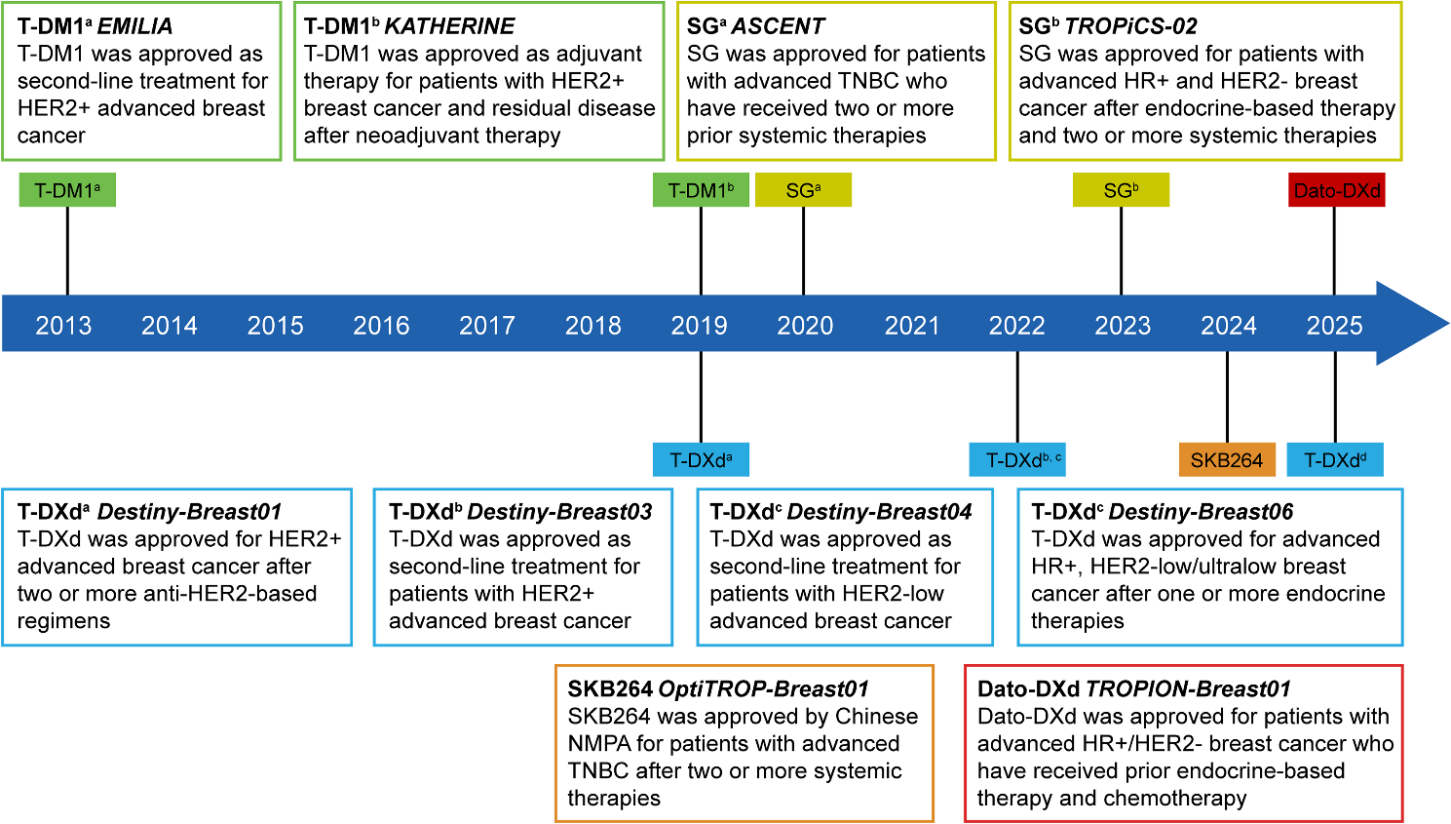
Timeline showing the approval of ADCs in breast cancer. Data cutoff date, January 31, 2025. T-DM1, trastuzumab emtansine; T-DXd, trastuzumab deruxtecan; SG, sacituzumab govitecan; Dato-DXd, datopotamab deruxtecan; HER2, human epidermal growth factor receptor 2; HR, hormone receptor; TNBC, triple-negative breast cancer; NMPA, national medical products administration; -/+, negative/positive

**Fig. 3:**
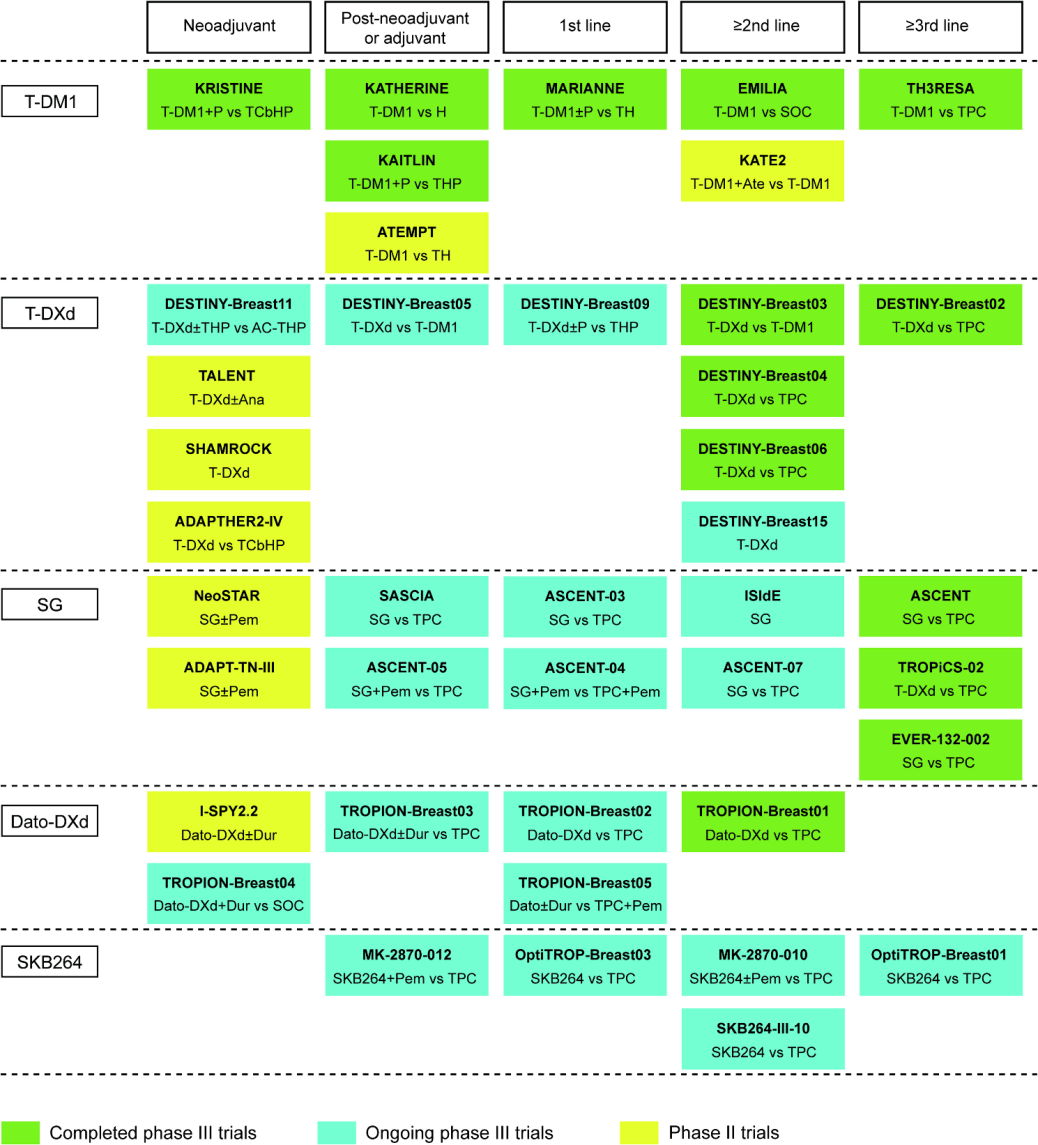
Pivotal clinical trials of ADCs in breast cancer according to different agents and treatment settings. Data cutoff date, January 31, 2025. T-DM1, trastuzumab emtansine; T-DXd, trastuzumab deruxtecan; SG, sacituzumab govitecan; Dato-DXd, datopotamab deruxtecan; P, pertuzumab; TCbHP, docetaxel-carboplatin-trastuzumab-pertuzumab; H, trastuzumab; THP, taxane-trastuzumab-pertuzumab, TH, paclitaxel-trastuzumab; SOC, standard of care; TPC, treatment of physician’s choice; Ana, anastrozole; Ate, atezolizumab; AC, anthracycline-cyclophosphamide; Pem, pembrolizumab; Dur, durvalumab

The timeline showing the approval of ADCs in breast cancer is illustrated in Fig. 2. Pivotal clinical trials of ADCs in breast cancer according to different agents and treatment settings are shown in Fig. 3. Currently, there are more than 150 ADCs that are being actively investigated in clinical trials [11, 12], and more promising data will emerge.

### Future directions

**Fig. 4:**
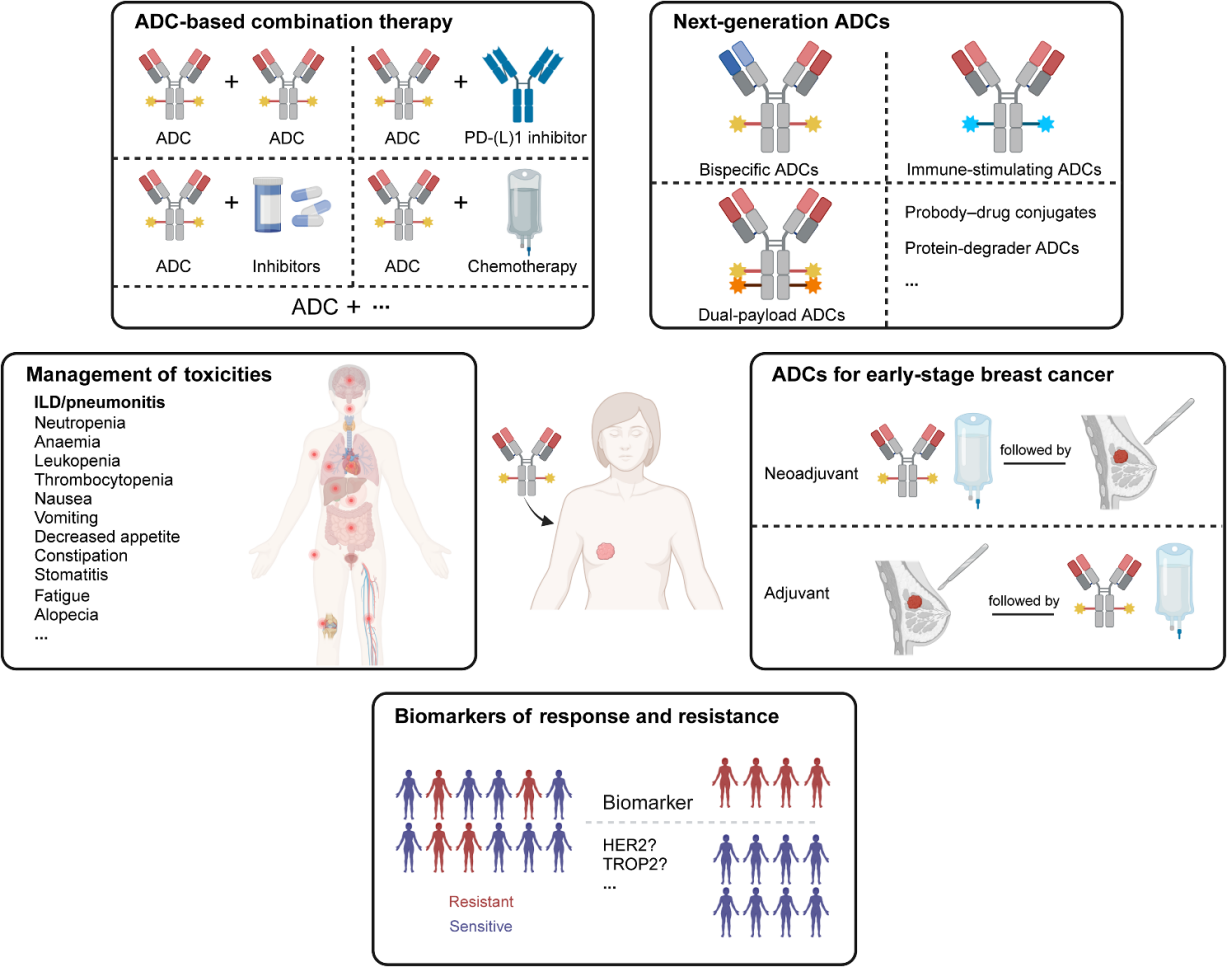
Future directions of ADCs in breast cancer. ADC, antibody–drug conjugate; HER2, human epidermal growth factor receptor 2; TROP2, trophoblast cell surface antigen-2; ILD, interstitial lung disease; PD-(L)1, programmed death-1/programmed death-ligand 1. Created in BioRender. Li, N. (2025) https://BioRender.com/t70c288

Despite the current success of ADCs, there is still a lot that needs to be further investigated to optimize ADC therapy. In the rapid pace of ADC development, several notable future directions of ADC research are described below (Fig. 4).

### ADC-based combination therapy

Despite the promising activity of ADCs in solid tumors, the benefit of ADCs as a single agent is limited as a result of the emergence of resistance. In theory, combination therapies can overcome resistance to both drugs. Thus, ADC-based combinations with other antitumor therapies including immunotherapy, targeted therapy, and chemotherapy are being evaluated in clinical trials. Of note, the combinations of ADCs with other therapies should balance the optimal efficacy and safety. The ideal combination partners for ADCs may provide synergistic antitumor effects without unacceptable overlapping toxicities.

For cisplatin-ineligible advanced urothelial cancer, the combination of an anti-Nectin-4 ADC enfortumab vedotin and an anti-PD-1 antibody pembrolizumab led to an encouraging OS and PFS result as first-line treatment, resulting in the approval of enfortumab vedotin plus pembrolizumab for such patients [98]. The KATE2 trial comparing T-DM1 plus atezolizumab with T-DM1 plus placebo failed to show improvement in PFS with T-DM1 plus atezolizumab. However, a trend towards an improvement in PFS was observed in the subgroup of patients with positive expression of PD-L1 (median PFS 8.5 vs. 4.1 months, *P* = 0.099), suggesting that the addition of an anti-PD-1/PD-L1 antibody to HER2-targeting ADC might be of benefit in the PD-L1 positive population [30]. For advanced TNBC, the BEGONIA trial investigated the combination of Dato-DXd and durvalumab as first-line treatment, and showed encouraging efficacy, with an ORR of 79% [71]. For localized breast cancer, in the I-SPY2.2 trial, neoadjuvant single-agent Dato-DXd led to a pCR rate of about 30% for TNBC and less than 10% for HR+/HER2- breast cancer [99], and the combination of Dato-DXd and durvalumab as neoadjuvant therapy resulted in a pCR rate of 46% for TNBC, and 21% for HR+/HER2- breast cancer [100]. These preliminary data indicated that the addition of an immune checkpoint inhibitor to an ADC may act synergistically.

The FB-10 trial revealed that T-DM1 plus an anti-HER2 tyrosine kinase inhibitor (TKI) neratinib led to an ORR of 63% in patients with metastatic HER2-positive breast cancer [101]. The synergistic effects of T-DM1 and neratinib were also shown in the phase II TBCRC 022 trial [102]. T-DM1 can also synergize with a TKI lapatinib in the neoadjuvant setting [103]. For HER2-positive metastatic breast cancer, T-DM1 may act in concert with cyclin-dependent kinase 4 and 6 (CDK 4/6) inhibitors [104, 105] and phosphoinositide 3-kinase (PI3K) inhibitors [106]. The results of the MARIANNE trial indicated that the first-line treatment of T-DM1 with pertuzumab did not show its superiority in survival outcomes compared with T-DM1 alone and the standard trastuzumab-plusa-taxane regimen [31, 107]. The phase Ib SEASTAR study showed that poly(ADP-ribose) polymerase (PARP) inhibitor rucaparib plus SG has a promising antitumor activity [108]. Multiple clinical trials assessing the combination of ADCs with other agents for advanced breast cancer are currently ongoing, including Destiny-Breast 07 (NCT04538742), Destiny-Breast 08 (NCT04556773), Destiny-Breast 09 (NCT04784715), HER2CLIMB-04 (NCT02614794), HER2CLIMB-02 (NCT03975647), ASCENT-04 (NCT053822860), ASSET (NCT05143229), TROPION-Breast05 (NCT06103864), and MK-2870-010 (NCT06312176).

### Management of toxicities

Given the rapid expansion of ADCs in their indications, an awareness of AEs and the management of toxicities are crucial. The toxicities of ADCs often consist of on-target and off-target toxicities, with the latter dominating the toxicity profiles of most ADCs. Data from a meta-analysis showed that the incidence of all-grade treatment-related adverse events was 91.2%, and the grade ≥ 3 AEs was 46.1% [109]. The main toxicities may vary across different ADCs. For example, the most common grade ≥ 3 AEs of T-DM1 were thrombocytopenia (13%), elevated AST (4%), elevated ALT (3%), anemia (3%), fatigue (2%), hypokalaemia (2%), neutropenia (2%), and diarrhea (2%) [10]. In contrast, the most common grade ≥ 3 AEs of T-DXd were neutropenia (19%), anemia (14%), leukopenia (10%), thrombocytopenia (7%), nausea (5%), fatigue (4%), vomiting (2%), and interstitial lung disease (ILD)/pneumonitis (2%) [110].

Concern has been raised regarding the lung toxic effects of ADCs since deaths related to ILD/pneumonitis have been reported. Lung toxicity was particularly more frequent with T-DXd. A comprehensive summary of T-DXd suggested the incidence of ILD/pneumonitis of any grade and grade ≥ 3 was 12.5% and 2.2%, respectively [110]. One possible mechanism of ILD is the payloads of ADCs. Payloads potentially inducing ILD include maytansinoids (emtansine), auristatins, camptothecins (deruxtecan), and duocarmycins. Another possible mechanism of ADC-induced ILD/pneumonitis was target-independent uptake of the conjugates by alveolar macrophages [111]. The proper management of ADC–related ILD/pneumonitis includes early diagnosis, careful monitoring, and appropriate treatment [112]. Although ADC-induced ILD/pneumonitis can be life-threatening, most cases are low-grade and can be resolved upon in-time corticosteroid treatment.

Several strategies have been pursued to optimally manage ADC-associated toxicities, including optimizing the dose and treatment schedule, close monitoring, early diagnosis, and preventative drug use. In addition, novel ADCs with high efficiency and low toxicity, and preclinical and translational studies of the mechanisms of ADC-associated toxicities are worth exploring.

### Biomarkers of response and resistance

Despite the encouraging results of ADCs in breast cancer, like other antitumor agents, intrinsic and acquired resistance emerges during the use of ADCs. Thus, exploring potential biomarkers of response and resistance is needed. ADCs consist of three main components: a monoclonal antibody, a cytotoxic payload, and a chemical linker. The resistance mechanisms to ADCs may be relatively complex since the ADC part(s) responsible for the emergence of resistance should be identified. These mechanisms of resistance include antibody-related resistance, payload-related resistance, and linker-related resistance.

Repeated collection of tumor samples is crucial to identifying possible biomarkers of response and resistance. For HER2-directing ADCs, higher HER2 expression is associated with enhanced T-DM1 efficacy in a phase II trial [113]. HER2 heterogeneity is reported to be a predictor of resistance to T-DM1 plus pertuzumab in the neoadjuvant setting in a phase II study [114]. HER2 expression was identified as a determinant of T-DXd efficacy in the DAISY trial, with the ORRs being 70.6%, 37.5%, and 29.7% for HER2-overexpressing, HER2-low, and HER2-zero tumors [48]. Of note, results from the Destiny-Breast04 trial showed the high efficacy of T-DXd in HER2-low breast cancer [46]. These results indicate that other molecular mechanisms contribute to the effectiveness of ADCs, and there are other biomarkers of response. Indeed, the level of HER2 gene amplification predicts a pathological complete response in HER2-positive breast cancer, and OS in HER2-positive gastric cancer [115, 116].

For TROP2-targeting ADCs, TROP2 was identified as a response determinant and a mechanism of resistance for SG in TNBC, with one patient showing intrinsic resistance to SG and lacking TROP2 expression and another patient with TACSTD2 (encoding TROP2) mutation having acquired resistance to SG [117]. However, higher expression of TROP2 was not associated with better efficacy from the biomarker analyses of the ASCENT trial [59]. The ongoing phase II ICARUS-BREAST01 trial is evaluating HER3-DXd in patients with advanced HR+/HER2- breast cancer. Within the trial, the patients underwent tumor biopsies at baseline, on treatment and at the end of treatment, thus facilitating the exploration of potential biomarkers of response and resistance to ADCs [118].

Preclinical and translational studies showed that the reduction of HER2 expression decreases the efficacy of T-DM1 [119], high expression of RAB5A is associated with increased efficacy of T-DM1 [120], impaired lysosomal proteolytic activity contributed to resistance to T-DM1 [121], loss of immunogenic cell death resulted in resistance of T-DM1 [122], targeting TACC3 induces immunogenic cell death and overcomes resistance to T-DM1 [122], targeting EGFR overcomes resistance to T-DXd [123], and DNA repair pathways was associated with HER2-directing ADC (T-DM1 and T-DXd) resistance [124]. Detailed reviews for biomarkers of response and resistance were reported elsewhere [125, 126]. However, more research is still needed to identify the biomarkers of response and resistance since most ADCs (except for T-DM1) for breast cancer have not entered clinical practice for a long time.

### ADCs for early-stage breast cancer

T-DM1 has been established as adjuvant therapy for patients with non-pCR disease after neoadjuvant therapy for HER2-positive breast cancer based on the results from the KATHERINE trial in 2019 [32]. The attempt to replace trastuzumab with T-DM1 in the dual anti-HER2 regimens as neoadjuvant or adjuvant treatment was unsuccessful. In the KRISTINE trial comparing T-DM1-pertuzumab and TCbHP as neoadjuvant therapy for HER2-positive stage II–III operable breast cancer, T-DM1-pertuzumab was associated with a lower pCR rate than TCbHP (44.4% vs. 55.7%) [33]. The KAITLIN trial AC-KP (T-DM1-pertuzumab) was found to have no significant iDFS advantage compared to AC-THP as adjuvant therapy [34]. Adjuvant T-DM1 for 1 year provided a favorable 5-year iDFS for stage I HER2-positive breast cancer in the ATTEMPT trial [35, 36]. The DESTINY-Breast05 trial (NCT04622319) is currently ongoing to challenge T-DM1 with T-DXd as adjuvant therapy for patients without pCR after neoadjuvant therapy. In the neoadjuvant setting, the DESTINY-Breast11 trial (NCT05113251) is evaluating whether the T-DXd alone or T-DXd followed by THP can replace the standard TCbHP regimen.

The phase III SASCIA trial is investigating the efficacy and safety of SG in patients with HER- breast cancer with non-pCR disease after neoadjuvant therapy. The interim safety analysis showed that the safety profile of SG was manageable [66]. In the phase II NeoSTAR trial, the preliminary results showed that neoadjuvant SG alone for patients with early-stage TNBC resulted in a pCR rate of 30% [67]. In the I-SPY2.2 trial, the pCR rate after neoadjuvant Dato-DXd alone is less than 10% for HR+/HER2- disease and about 30% for TNBC [99]. The combination of Dato-DXd and durvalumab as neoadjuvant therapy in the I-SPY2.2 trial resulted in a pCR rate of 21% for HR+/HER2- breast cancer and 46% for TNBC [100]. The TROPION-Breast04 trial (NCT06112379) is currently ongoing to investigate the efficacy and safety of neoadjuvant Dato-DXd and durvalumab versus standard pembrolizumab plus chemotherapy (the KEYNOTE-522 regimen [127]) for early-stage TNBC and HR-low/HER2- breast cancer. The ongoing phase III TROPION-Breast03 trial is evaluating Dato-DXd with or without durvalumab versus the standard of care as adjuvant therapy in patients with stage I–III TNBC and residual invasive disease after neoadjuvant therapy [128].

### Exploring ADCs of next generation

Major impediments that arise during the use of ADCs include acquired resistance and toxicities. As linker technology has substantially advanced over the past two decades, the design of ADCs has had considerable improvements. Next-generation ADCs including bispecific ADCs, probody–drug conjugates, immune-stimulating antibody conjugates, and dual-payload ADCs aiming to improve efficacy and safety, are currently being investigated in the treatment of malignancies. These novel ADC formats have unique features that aim to enhance efficacy, decrease toxicities, and deal with resistance and heterogeneity.

The biparatopic ADC MEDI4276 which targets two distinct HER2 epitopes in extracellular domains 2 and 4 did not display a good activity-toxicity balance for patients with breast cancer [82]. Another biparatopic ADC zanidatamab zovodotin (ZW49) which binds to extracellular domains 2 and 4 of HER2 had shown a manageable safety profile and encouraging antitumor activity in solid cancers, with a confirmed ORR of 28% [129]. The EGFR-HER3 bispecific ADC BL-B01D1 was associated with an ORR of 60% in solid tumors with an acceptable safety profile [130]. In a phase I/II clinical trial evaluating a probody–drug conjugate praluzatamab ravtansine (CX-2009) in advanced solid tumors, among 22 patients with HR+/HER2- breast cancer, 2 (9%) had PR, and 10 (45%) had SD [97]. A HER2-targeting immune-stimulating antibody conjugate BDC-1001 with a TLR7/8 agonist payload was well tolerated and displayed encouraging clinical activity in HER2-expressing tumors [131]. There are a wide variety of next-generation ADCs that are being tested in preclinical and clinical studies. The rapid development of next-generation ADCs will offer more options for the treatment of malignancies.

## Conclusions

ADCs have become a revolutionary treatment modality in tackling cancer. For breast cancer, T-DM1, T-DXd, SG, Dato-DXd, and SKB264 have been approved for the treatment of breast cancer, and multiple agents are in late-stage clinical development. The successful development of ADCs is shaping the treatment standard of care for breast cancer. A better understanding of the design and development of ADCs will promote reasonable clinical use and rational design of clinical trials. However, several issues have arisen during the use of ADCs, including acquired resistance and toxicities. Future studies for ADCs should focus on several directions, including identification of biomarkers for response, clarification of mechanisms of resistance, exploration of rational combination regimens, and design of superior next-generation ADCs.

## Data Availability

No datasets were generated or analysed during the current study.

## Abbreviations

ADCs: Antibody-drug conjugates
ADCC: Antibody-dependent cellular cytotoxicity
ADCP: Antibody-dependent cellular phagocytosis
AEs: Adverse events
CDC: Complement-dependent cytotoxicity
CDK 4/6: Cyclin-dependent kinase 4 and 6
CNS: Central nervous system
CR: Complete response
DAR: Drug-to-antibody ratio
Dato-DXd: Datopotamab deruxtecan
DXd: Deruxtecan
HER2: Human epidermal growth factor receptor 2
HR: Hormone receptor
iDFS: Invasive disease-free survival
IgG: Immunoglobulin G
IHC: Immunohistochemistry
ISH: In situ hybridization
ILD: Interstitial lung disease
M1S1: Membrane component chromosome 1 surface marker 1
MMAE: Monomethyl auristatin E
MMAF: Monomethyl auristatin F
ORR: Objective response rate
OS: Overall survival
PARP: Poly(ADP-ribose) polymerase
pCR: Pathologic complete response
PD-1: Programmed death-1
PD-L1: Programmed death-ligand 1
PFS: Progression-free survival
PI3K: Phosphoinositide 3-kinase
PR: Partial response
SD: Stable disease
SG: Sacituzumab govitecan
TAC-STD2: Tumor-associated calcium signal transducer 2
TCbHP: Docetaxel-carboplatin-trastuzumab-pertuzumab
T-DM1: Trastuzumab emtansine
T-Duo: Trastuzumab duocarmazine
T-DXd: Trastuzumab deruxtecan
THP: Taxane-trastuzumab-pertuzumab
TKI: Tyrosine kinase inhibitor
TNBC: Triple-negative breast cancer
TROP2: Trophoblast cell surface antigen-2
ZW49: Zanidatamab zovodotin
